# 

**Published:** 1869-10

**Authors:** 


### CONTENTS.

ORIGINAL COMMUNICATIONS.

Dental Ethics ..... 405
Miscellanies..... 413
On the Renouncing of Rubber by the Dental Profession..... 422
Alarming Hemorrhage..... 429

SELECTIONS.

Spectacles or No Spectacles..... 430
A Feigned Tumor of the Jaw..... 431
Disarticulation of the Upper Maxillary Bones..... 432
On the Medicinal Use of Phosphorous and its Compounds..... 434
Use of Chloride of Gold..... 436
The Highest Branch of Medicine..... 437
Detection of Blood Stains..... 438
Chloral—A New Anæsthetic..... 438
New Operation in Dental Science..... 440
Hypertrophy of Nails and Bone ..... 442
Organic Synthesis..... 442
Chromic Acid..... 442

EDITORIAL.

Use of Os-Artificial..... 443
Worse than the Wicked Flea..... 445
Educational..... 447

